# Supplementary material for: Investigating the influence of physiologically relevant hydrostatic pressure on CHO cell batch culture
Source: Sci Rep. 2021 Jan 8;11:162. doi: 10.1038/s41598-020-80576-8 (PMC7794228; doi:10.1038/s41598-020-80576-8)
Supplement: Supplementary file 1 — Supplementary Information 1. [file 41598_2020_80576_MOESM1_ESM.zip › S1/IgG/Feb14 0 30 60 90/60mmHg Feb6 2.pdf]

Data File C:\Users\P...emStation\1\Data\TK_CHO_IgG 2019-02-14 14-17-55\044-11-ML 60 Feb6.D
Sample Name: ML 60 Feb6

===

Acq. Operator : SYSTEM Seq. Line : 44
Sample Operator : SYSTEM
Acq. Instrument : HPLC-0039 Location : 11
Injection Date : 2/14/2019 5:23:52 PM Inj : 1
Inj Volume : 100.000 µl
Method : C:\Users\Public\Documents\ChemStation\1\Data\TK_CHO_IgG 2019-02-14 14-17-55\CHO_IgG_LC.M (Sequence Method)
Last changed : 2/8/2019 3:17:23 PM by SYSTEM

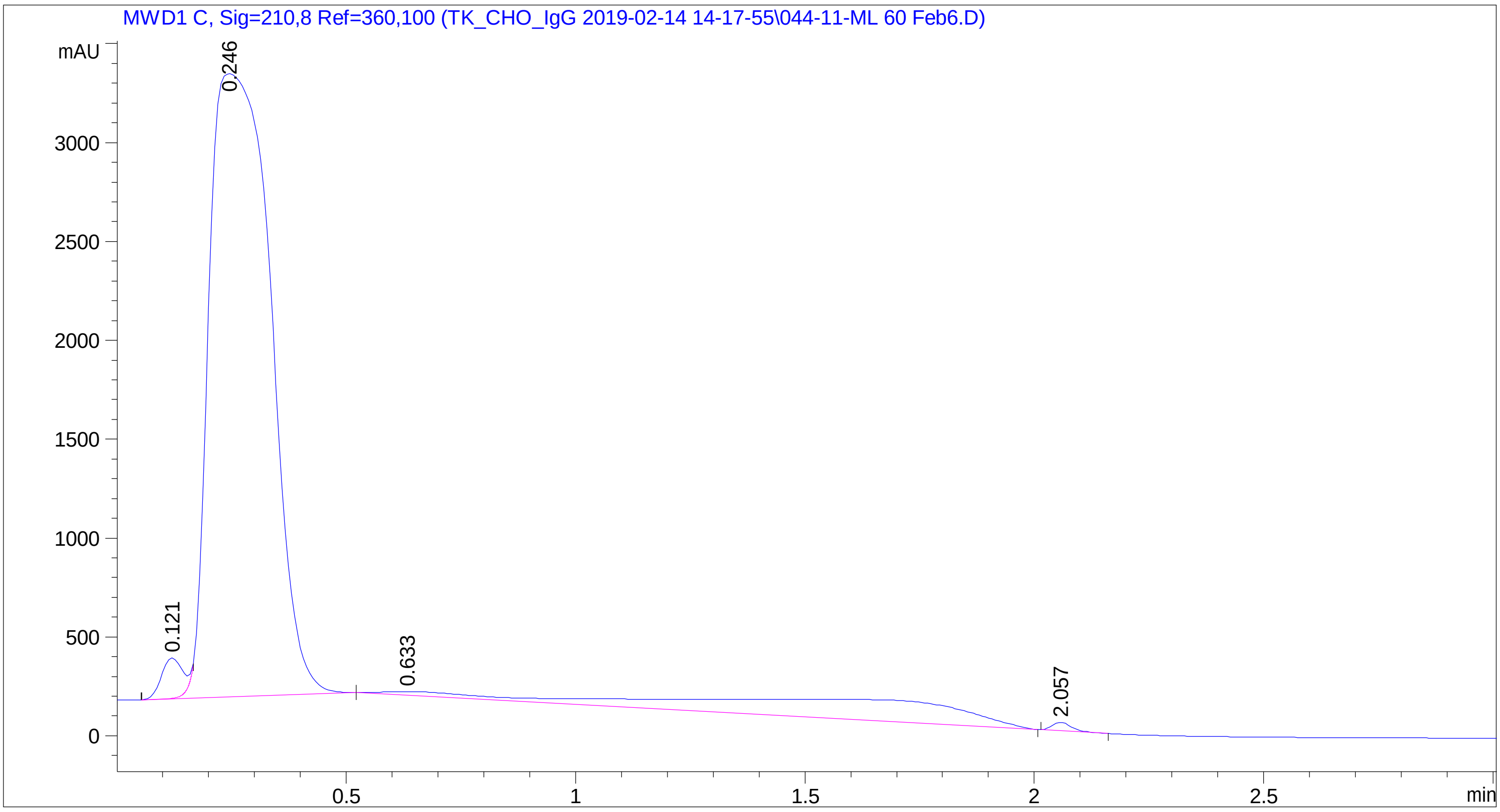

===

Area Percent Report

===

Sorted By : Signal
Multiplier : 1.0000
Dilution : 1.0000
Use Multiplier & Dilution Factor with ISTDs

Signal 1: MWD1 C, Sig=210,8 Ref=360,100

Peak #	RetTime [min]	Type	Width [min]	Area [mAU*s]	Height [mAU]	Area %
1	0.121	BV E	0.0485	638.26398	203.85542	1.8443
2	0.246	VB R	0.1521	2.91956e4	3150.59375	84.3612
3	0.633	BB	3.0517	4647.24023	18.07449	13.4283
4	2.057	BBA	0.0469	126.74294	42.35891	0.3662
Totals :				3.46079e4	3414.88257	

===

*** End of Report ***
